# The Role of microRNAs in Mitochondria: Small Players Acting Wide

**DOI:** 10.3390/genes5040865

**Published:** 2014-09-26

**Authors:** Filipe V. Duarte, Carlos M. Palmeira, Anabela P. Rolo

**Affiliations:** 1 Center for Neuroscience and Cell Biology, University of Coimbra, Coimbra 3004-504, Portugal; E-Mails: aprolo@ua.pt (A.P.R.); palmeira@ci.uc.pt (C.M.P.); 2 Department of Life Sciences, University of Coimbra, Coimbra 3000-456, Portugal; 3 Department of Biology, University of Aveiro, Aveiro 3810-193, Portugal

**Keywords:** metabolism, mitochondria, mitochondrial dynamics, miRNAS, OXPHOS

## Abstract

MicroRNAs (miRNAs) are short, single-stranded, non-coding RNA molecules that act as post-transcriptional gene regulators. They can inhibit target protein-coding genes, through repressing messenger RNA (mRNA) translation or promoting their degradation. miRNAs were initially found to be originated from nuclear genome and exported to cytosol; where they exerted most of their actions. More recently, miRNAs were found to be present specifically in mitochondria; even originated there from mitochondrial DNA, regulating in a direct manner genes coding for mitochondrial proteins, and consequently mitochondrial function. Since miRNAs are recognized as major players in several biological processes, they are being considered as a key to better understand, explain, and probably prevent/cure not only the pathogenesis of multifactorial diseases but also mitochondrial dysfunction and associated diseases. Here we review some of the molecular mechanisms purported for miRNA actions in several biological processes, particularly the miRNAs acting in mitochondria or in mitochondria-related mechanisms.

## 1. Introduction

MicroRNAs (miRNAs) are short, single-stranded, non-coding RNA molecules (19–23 nucleotides) that act by post-transcriptional modulation of protein-coding genes, through repressing messenger RNA (mRNA) translation or promoting their degradation. Each miRNA can affect multiple target genes. This makes them a potential key to understanding the pathogenesis of chronic multifactorial diseases, such as cardiovascular diseases, diabetes, obesity, and cancer [1,2].

miRNAs align and bind especially to 3'UTR sequences of their target genes and initiate either mRNA degradation or translational repression, resulting in reduced protein levels. miRNAs are now recognized as major players in almost every biological process such as cell proliferation, apoptosis, differentiation and organogenesis [3], and these molecules can also be transported between cells and tissues via circulation [4]. These circulating miRNAs have been shown to participate in cell-to-cell communication [5], potentially contributing to disease progression.

In recent years, the discovery of miRNAs has revolutionized the traditional view of gene expression and our understanding of miRNA biogenesis and function has expanded. It is more than clear that their abnormal expression may result in and modulate pathophysiological processes in the cell and, consequently, lead to the development of many diseases. Hopefully, a better understanding of miRNAs biology and functions will not only provide better knowledge about molecular mechanisms of disease, but also give rise to new therapeutical approaches.

Here we review some of the molecular mechanisms purported for miRNA actions, particularly the miRNAs acting in mitochondria or in mitochondria-related pathways. The involvement of miRNAs in mitochondrial metabolism, mitochondrial oxidative phosphorylation (OXPHOS), electron transport chain (ETC) components, lipid metabolism and metabolic disorders will be addressed, as well as miRNAs contribution for mitochondrial dynamics, aging and apoptosis regulation.

## 2. Mitochondria

Mitochondria are cellular organelles delimited by two membranes that embrace about one tenth of the cell’s proteins. The mitochondrion consists of four main structures or compartments: two membranes, the intermembrane space and the matrix within the inner membrane.

The mitochondrial outer membrane (MOM) separates the cytosol from the intermembrane space. The MOM is responsible for interfacing with the cytosol and its interactions with cytoskeletal elements, which are important for the movement of mitochondria within a cell. This mobility is essential for the distribution of mitochondria during cell division and differentiation. The mitochondrial inner membrane (MIM) separates the intermembrane space from the matrix. The folding of the MIM (cristae) serves to increase the surface area of this membrane. The MIM hosts the most important redox reactions converting the energy of nutrients into ATP. These reactions are catalyzed by the mitochondrial ETC, which transports electrons from several substrates to oxygen, in the complex multistep process termed mitochondrial respiration. According to the chemiosmotic theory, mitochondrial respiration generates a transmembrane potential (ΔΨm) across the inner membrane, which is used by ATP synthase to phosphorylate ADP. The MIM is normally impermeable to protons and other ions, and this solute barrier function of the MIM is critical for energy transduction. Permeabilization of the MIM dissipates ΔΨm and thereby uncouples the process of respiration from ATP synthase, halting mitochondrial ATP production [6].

In addition to the process of ATP formation, mitochondria are highly dynamic organelles that have been implicated in the regulation of a great and increasing number of physiological processes. Mitochondrial function is a key to cell life and death, and the deregulation of mitochondrial metabolism is critical to the pathogenesis of several diseases. Cells need energy not only to support their vital functions but also to die gracefully, through programmed cell death, or apoptosis [6]. Furthermore, mitochondrial regulation is also present beyond cell death mechanisms. Indeed, besides oxidative ATP production, mitochondria assume other functions such as heme synthesis, β-oxidation of free fatty acids, metabolism of certain amino acids, production of free radical species, formation and export of Fe/S clusters, iron metabolism, and play a crucial role in calcium homeostasis [7]. Still, the regulatory roles of mitochondria over normal physiology include the transduction pathway that underlies the secretion of insulin in response to glucose by β-cells. The physiological “uncoupling” of mitochondria also plays a central role as a heat-generating mechanism in non-shivering thermogenesis in young mammals. It has also been suggested that the production of free radical species by mitochondria may play a key role as a signaling mechanism, for example, in the regulation of ion-channel activities and also in initiating cytoprotective mechanisms in stressed cells [7].

Mitochondrial OXPHOS produces more than 95% of a cell’s energy in the form of ATP under normal physiological conditions. This process involves five different protein complexes, Complex I-V. The respiratory chain or electron transport chain (ETC; Complexes I–IV) in the MIM is associated with electron transfer components—coenzyme Q and cytochrome *c*. In the matrix, pyruvate oxidation, β-oxidation of fatty acids, and the tricarboxylic acid cycle (TCA cycle) pathways are associated.

The total number of polypeptides involved directly in OXPHOS is 91 when cytochrome *c* is included. Of these proteins, some are nuclear-encoded, and some are mitochondrial-encoded in the mtDNA [8]. Because of its limited coding capacity mtDNA relies on nuclear genes for structural components and biological functions. Besides, nuclear-encoded genes also regulate mitochondrial transcription, translation, and mtDNA replication, thus the precise cooperation of nuclear and mtDNA expression is essential to regulate OXPHOS capacity in response to different physiological demands and disease states [9,10]. Dysregulated mtDNA expression has been associated with human mitochondrial diseases and is also observed in normal aging process [11,12]. The overall process of oxidative phosphorylation is tightly controlled by transcriptional regulation at the level of DNA, translational effects via RNA levels and stability, by substrate feedback inhibition, and by post-translational modifications, including phosphorylation and acetylation. Inefficient electron transfer through complexes I-IV causes human disease in part because of loss of energy metabolism but also because insults to the various enzymes (particularly Complexes I, II and III) induce production of toxic reactive oxygen species (ROS). Defects of complex V are also a cause of mitochondrial dysfunction [13]. It has also been reported that the deterioration of mitochondrial function underlies common metabolic-related diseases [14,15], and several studies have identified compromised oxidative metabolism, altered mitochondrial structure and dynamics, and impaired biogenesis and gene expression in insulin resistance or type 2 diabetes (T2DM) models [16,17].

Mitochondria also play a pivotal role in the process of cell death. It has been generally accepted that cells die by necrosis when ATP is not sufficient, or they die by the process of apoptosis when sufficient ATP is available. Apoptosis (programed cells death) is a process coordinated by a family of proteases—the caspases, which participate in the molecular control of apoptosis as triggers of cell death and as regulatory elements within this process. Excessive accumulation of Ca^2+^ leads to the formation of ROS and to opening of the mitochondrial permeability transition pore (mPTP), which depolarizes the mitochondria and leads to mitochondrial swelling. This may also provide a mechanism for the release of cytochrome *c* from the intermembrane space into the cytoplasm. Cytochrome *c* normally functions as a part of the respiratory chain, but when released into the cytosol it becomes a critical component of the apoptosis execution machinery, where it activates caspases and causes apoptotic cell death. Apoptosis may be triggered by extracellular signals (extrinsic pathway) or by intracellular processes (intrinsic pathway) [18]. An increased mitochondrial formation of ROS triggers the intrinsic pathway by opening permeability transmission pores with increased permeability of the outer mitochondrial membrane.

Mitochondrial shape is very heterogeneous, ranging from small spheres to interconnected tubules. During cell life, mitochondria undergo continuous cycles of fusion and fission. Therefore, mitochondria are now recognized as highly dynamic organelles that move throughout a cell and constantly fuse and divide [19]. Studies over the last several years indicate that these membrane-remodeling processes promote homogenization of the mitochondrial population by content mixing and thereby preserve mitochondrial function. It was reported, in pancreatic β-cells, that fusion and fission events are paired. Fusion triggers fission, but fission has no effect on the following fusion event [20]. Research on mitochondrial fusion and fission (collectively termed mitochondrial dynamics) gained much attention in recent years, as it is important for our understanding of many biological processes, including the maintenance of mitochondrial functions, apoptosis and ageing [21].

## 3. Translocation of miRNAs to Mitochondria

The discovery of mitochondrial-located miRNAs (mitomiRs) raises the issue of the molecular mechanism underlying their translocation from the nucleus to the mitochondria. Studies in different species indicate that it may exist a number of import pathways of nucleus-encoded RNAs to mitochondria, being the most of them largely ATP-dependent. Nonetheless, the molecular mechanisms of mitochondrial RNA import seem to vary largely in a species-specific way [22].

It has been reported that pre-miRNAs, as well as mature miRNAs, are present in the mitochondria, and these findings have also raised the possibility of mitochondrial miRNA synthesis [23]. It was suggested that some pre-miRNAs sequences seem to be processed in the mitochondria giving origin to mature miRNAs, which could be immediately active on the mitochondrial transcripts or exported to the cytosol in order to interfere with genomic-derived mRNA. Thus, the mitochondrial-processed miRNAs are likely to contribute to some post-transcriptional regulation of gene expression related to the mitochondrial functions [24].

Coming from their location, the mitochondria, some miRNAs are currently named as mitomiRs; it refers to those miRNAs that can localize in mitochondria whether transcribed from the nuclear or, potentially, the mitochondrial genome. When their genomics was analyzed, a number of mitomiRs mapped the nuclear genome at loci relevant to mitochondrial functions or diseases [22]. Moreover, the set of mitomiRs may vary depending on the cell type. Indeed, a targeted approach was already used by customizing tissue-specific miRNA microarrays, which led to the identification of rat cardiac-specific mitomiRs, namely miR-181c [25].

The identification of populations of miRNAs in the mitochondria pushed scientists in the field to question its biological functions. It is established that miRNAs, originated in the nuclear genome, are exported to cytosol where they are processed and exert their function by inhibiting nuclear genome-derived mRNA. Actually it is also known that some miRNAs are imported into mitochondria where they interact with some mitochondrial genome-derived mRNA molecules. More strikingly, it has also come to light that mitochondrial genome (mtDNA) can originate some miRNA molecules that exert their function directly on mitochondrial transcripts [22].

Actually, apart from the presence in the cytosol of miRNA regulating mRNAs that encode proteins involved in mitochondria-related activities, a small number of miRNAs were reported to be present within the mitochondrial matrix itself [26,27], with revealed apoptosis/cell-death and cell-cycle/cell-division as among the most significant processes targeted, but only one possible interaction with mtDNA-derived mRNA (between miR-130a and cytochrome *c* oxidase III) [26]. More recently, unique sets of mitochondrial miRNAs, or “mitomiRs,” were described for human skeletal muscle and HeLa cells [28,29]. The mitomiRs were found to have thermodynamic features and size distinct from canonical miRNAs and to be expressed almost entirely from nuclear genes in loci relevant to mitochondrial function. MitomiRs appeared to lack preferential targeting of nuclear-encoded mitochondrial genes when compared with a set of cytosolic miRNAs, but most were predicted by RNA22, RegRNA, miRWalk, or TargetScan algorithms to target multiple mtDNA sites, including many within all the mtDNA-encoded protein genes. Interestingly, human mtDNA also seems to harbor mitomiR sequences (namely, miR-1974, miR-1977, and miR-1978), but it remains to be ascertained whether miRNAs are actually transcribed from these mitochondrial genes [28].

## 4. Mitochondrial Modulation through miRNAs

### 4.1. Energy Metabolism

The location of miRNAs within the mitochondrion establishes an impressive intraorganellar mechanism for the fine-tuning of function to the metabolic demands of an organ or cell type. miRNAs have been widely studied in cancers, cardiovascular diseases, cholesterol metabolism, and diabetes [30,31,32].

Among the several roles that mitochondria play, energy metabolism is one of the most studied. As such, some miRNAs have already been identified as regulators in mitochondrial metabolism. miRNA-33 in particular has been shown to have a crucial role in cholesterol metabolism and also a conceivable use in clinical approaches [33]. Interestingly, some other miRNAs have been connected to lipid metabolism; miR-143 and -24 have been associated to increased incidence of Metabolic syndrome through polymorphisms in miRNA binding sites in APOL6 3'UTR [34]. In addition, miR-24 and miR-126 have been associated to fatty acid metabolism [35], whereas miR-204-5p associates with the oxidation of fatty acids [36]. miRNAs that are involved in metabolism and metabolic disorders indicate an important regulatory role in the key organs involved in lipid and glucose metabolism [37]. Accordingly, certain serum circulating miRNA profiles have been shown to be dysregulated in obese children [38], putting forward the idea that some serum miRNAs could be used as a potential predictive tool for obesity and type 2 diabetes [39].

**Figure 1:**
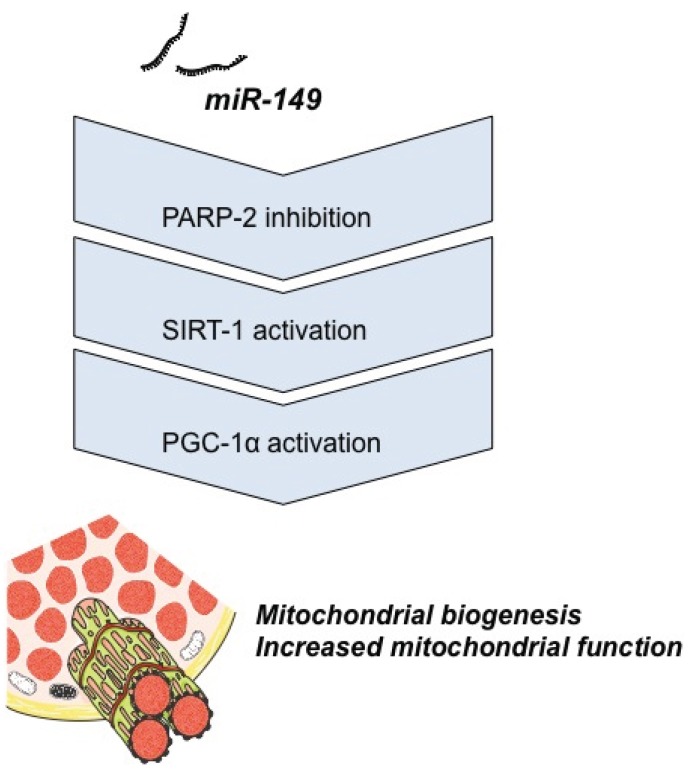
Schematic representation of the action of miR-149 in skeletal muscle mitochondrial biogenesis, via sirtuin-1 (SIRT-1)/peroxisome proliferator–activated receptor γ coactivator-1a (PGC-1α) axis. In obese mice, miR-149 is downregulated and mitochondrial function is reduced in the muscle.

High-fat diet (HFD) plays a central role in the initiation of mitochondrial dysfunction that significantly contributes to skeletal muscle metabolic disorders in obesity. Given the emerging roles of miRNAs in the regulation of skeletal muscle metabolism, some authors started to study miRNAs involvement in mitochondrial function/dysfunction related to metabolic disorders. Certain studies addressed whether activation of a specific miRNA pathway would rescue the HFD-induced mitochondrial dysfunction via the sirtuin-1 (SIRT-1)/ peroxisome proliferator–activated receptor γ coactivator-1a (PGC-1α) pathway [40]. Interestingly, it was shown that miR-149 inhibits poly(ADP-ribose) polymerase-2 (PARP-2) and so increases cellular NAD+ levels and SIRT-1 activity, that subsequently increases mitochondrial function and biogenesis via PGC-1α activation, as it is depicted in Figure 1. Furthermore, skeletal muscles from HFD-fed obese mice exhibit low levels of miR-149 and high levels of PARP-2, and they show reduced mitochondrial function and biogenesis due to a decreased activation of the SIRT-1/PGC-1α pathway, suggesting that mitochondrial dysfunction in the skeletal muscle of obese mice may be because of, at least in part, miR-149 dysregulation [40].

Moreover, another particular report has shown that HFD and excess fatty acids (FFAs) can cause enteric neuronal cell damage [41], contributing to delayed intestinal transit. The mechanism involve decreased enteric neuronal cell viability through mitochondrial damage and ER stress, being miR-375 strongly implicated in mediating the detrimental effects of HFD (e.g., by downregulating the pro-survival protein Pdk1 translation) [41]. HFD rich in saturated FFA rises oxidative stress in many tissues, such as brain and gastric and intestinal mucosa [42]. Mitochondria, as the major source of ROS, are susceptible to FFA accumulation and the ROS-induced lipid peroxidation [43]. A recent study has showed a significant increase in mitochondrial SOD after palmitate exposure, suggesting a mitochondrial oxidative stress overload [41]. It was also reported a significant decrease in the mitochondrial quantity measured by cytochrome *c* oxidase IV protein level, as well as ultrastructural changes consistent with mitochondrial dysfunction, all of these effects being at least in part mediated by miR-375. Systemic injection of miR-375 inhibitor to HFD-fed mice prevented the development of detrimental effects of HFD on intestinal transit and enteric neurons, providing direct evidence for the role of miR-375 on modulating ER stress and mitochondrial function [41].

Since when miRNA emerged as a new class of epigenetic regulators of gene expression, there has been great interest in its role modulating metabolism and promoting the development and progression of metabolic disorders, namely diabetes-related chronic complications [44]. Abnormal levels of some miRNAs have been observed in the development of diabetic kidney disease [45]. Additionally, the development of diabetic nephropathy in rats has been linked to the regulation of NOX4 levels by miR-25, where NOX4 is a catalytic subunit of NADPH oxidase [46]. Furthermore, other studies have shown that, in db/db mice, the increased miR-21 levels halted the proliferation of mesangial cells and reduced the 24h urine albumin releasing rate [47].

A fundamental function of mitochondria is to produce ATP via OXPHOS to supply energy for a variety of cellular functions. Unsurprisingly, also this vital function in mitochondria is targeted by miRNAs. MiR-15b, miR-16, miR-195 and miR-424 have emerged as regulators of the ATP levels [48]. The overexpression of these miRNAs was shown to suppress ATP levels and affect mitochondrial integrity as well, by acting on their common target, the ADP-ribosylation factor-like 2 (ARL2) mRNA [49]. Additionally, upregulation of other miRNAs such as miR-15b or miR-195 has been reported to induce mitochondrial degeneration and ATP reduction in cardiomyocytes [48].

In mitochondria, the regulation of energy metabolism can take place in both Krebs cycle and OXPHOS. Relatively to Krebs cycle, or TCA cycle, there are several miRNAs reported to be regulating several steps within the cycle, such as miR-183 downregulating isocitrate dehydrogenase or miR-743a downregulating malate dehydrogenase, all pushing the metabolism to a more glycolytic status [24]. Currently, it is also known that some miRNAs modulate mitochondrial OXPHOS, through targeting several mitochondrial players involved in electron transfer and ATP production. In the Table 1 we present a summary of miRNAs already reported to act in mitochondria and directly target some components involved in mitochondrial ETC. For example, miR-338 modulates OXPHOS and the mitochondrial function, targeting cytochrome *c* oxidase subunit IV (COX IV) mRNA in neurons [50]. OXPHOS process takes place in two major steps both coupled in inner mitochondrial membrane: the oxidation of nicotinamide adenine dinucleotide, its reduced form (NADH), or flavine adenine dinucleotide, its hydroquinone form (FADH_2_), and the phosphorylation of ADP to form ATP [51]. Presently there are also several studies reporting the regulation of OXPHOS by miRNAs, particularly: miR-210 downregulating Complex III in mitochondria; miR-181c, miR-210 and miR-338 downregulating Complex IV; and miR-141 downregulating Complex V in the mitochondrial ETC [24]. In particular, some authors have already reported that the rat mitomiR miR-181c is involved in electron chain complex IV remodeling in cardiomyocytes [25]. Similarly to other reports on mitomiRs, rat cardiomyocyte mitochondria were found to harbor a unique miRNA expression pattern, with miR-181c enriched 2-fold in the mitochondrial fraction with respect to whole heart because of translocation of the miRNA into the organelles, for which cytochrome *c* oxidase subunit I (mt-COX1) mRNA was confirmed as a target for miR-181c [25]. The authors showed that miR-181c, derived from the nuclear genome, translocates to the mitochondria, and more importantly, regulates mitochondrial gene expression and affects mitochondrial function. Coordination of nuclear gene expression and mitochondrial gene expression is thus essential. It was proven that in cardiomyocytes miRNA can regulate mitochondrial gene expression, specifically that miR-181c binds to the 3'-end of the mRNA of a mitochondrial gene, mt-COX1, a subunit of complex IV of the respiratory chain, and initially results in a decrease in mt-COX1 protein, complex IV remodeling, and increased production of ROS [25]. More recently, an *in vivo* method for administration of miR-181c in rats was used, displaying reduced exercise capacity and signs of heart failure, by targeting the 3'-end of mt-COX1 [52]. The mRNA levels of mitochondrial complex IV genes in the heart, but not any other mitochondrial genes, were significantly altered with miR-181c overexpression, suggesting selective mitochondrial complex IV remodeling due to miR-181c targeting mt-COX1. Isolated heart mitochondrial studies showed significantly altered O2-consumption, ROS production, matrix calcium, and mitochondrial membrane potential in miR-181c-treated animals, showing that miRNA delivered to the heart *in vivo* can lead to cardiac dysfunction by regulating mitochondrial genes [52]. Moreover, other authors reported an important role for miR-181a in regulating the mitochondrial apoptotic pathway in cardiomyocytes challenged with oxidative stress [53]. The downregulation of miR-181a significantly inhibited H_2_O_2_-induced cellular apoptosis, ROS production, the increase in malondialdehyde (MDA) levels, the disruption of mitochondrial structure, and the activation of key signaling proteins in the mitochondrial apoptotic pathway. miR-181a/c may thus represent potential therapeutic targets for the treatment of cardiovascular diseases.

**Table 1:** MicroRNAs (MiRNAs) with reported action directly on mitochondrial electron transport chain (ETC) components.

miRNA	Target	Model	Reference
miR-181c	mt-COX1	Rat, cardiomyocytes	[25]
miR-338	COX IV	Rat, neurons	[50]
miR-210	ISCU1/2; COX 10	Human, placenta and fibroblasts	[54]

A proper vascular system is also vital in the human placenta, in which fetal growth is critically dependent on energy metabolism driving active exchange of nutrients. Placental oxygen levels are therefore vital, and chronic hypoxia during pregnancy impairs fetal growth [55]. A role for miRNA was also found for placental hypoxia altering mitochondrial ETC function [54]. In human placental cells cultured under different oxygen concentrations, mitochondrial respiration was measured, alongside with the levels of ETC complexes. Expression of HIF-responsive miR-210 was increased in hypoxic fibroblasts and high-altitude placentas, whilst expression of its targets, iron-sulfur cluster scaffold (ISCU) and cytochrome *c* oxidase assembly protein (COX10), decreased. Moreover, protein synthesis inhibition, a feature of the high-altitude placenta, also suppressed ETC complex protein levels, demonstrating that mitochondrial function is altered and energy metabolism compromised in hypoxic human placentas, with specific intervention of miR-210.

Regarding the involvement of mitochondria in energy metabolism and miRNA modulation is the newly described relationship between miR-26 and mitochondrial morphology in adipocytes. Adipose tissue contains thermogenic adipocytes (*i.e.*, brown and brite/beige) that oxidize nutrients at exceptionally high rates via nonshivering thermogenesis. Its recent discovery in adult humans has opened up new avenues to fight obesity and related disorders such as diabetes. miR-26 was found to be a key regulator of human white and brite adipocyte differentiation, being upregulated in early adipogenesis [56]. Intriguingly, miR-26a significantly induced pathways related to energy dissipation, shifted mitochondrial morphology toward that seen in brown adipocytes, and promoted uncoupled respiration by markedly increasing the hallmark protein of brown fat, uncoupling protein 1 (UCP1) [56]. MiR-26a transfection evoked an increase in UCP1+ adipocytes up to 50%. Ultrastructural analysis by transmission electron microscopy (TEM) further revealed a shift of mitochondrial morphology toward brown adipocyte characteristics as well as a slightly increased density of cristae. Additionally, mitochondria of adipocytes from miR-26a-transfected cells were bigger in size and more roundish (*i.e.*, brown-like) in the late brite stage, indicating that miR-26a/b promote characteristics of energy-dissipating thermogenic adipocytes during human adipocyte differentiation. Additionally, prohibitin (PHB) has also been reported to play a crucial role in adipocyte differentiation and mitochondrial function, a process also modulated by miRNA action [57]. The levels of both miR-27a and miR-27b were shown to be downregulated following adipogenic induction of human adipose-derived stem cells, whereas the mRNA level of PHB was upregulated. Moreover, overexpression of miR-27a or miR-27b inhibited PHB expression and adipocyte differentiation. More specifically, it was shown that ectopic expression of miR-27a or miR-27b impaired mitochondrial biogenesis, structure integrity, and Complex I activity accompanied by excessive ROS production [57], lightening an anti-adipogenic role of miR-27.

### 4.2. Mitochondrial Dynamics

Having not always been like that, mitochondria are nowadays known to be highly dynamic organelles that possess two opposing activities. They divide and fuse constantly, and the balance between mitochondrial fission and fusion affects the morphology of mitochondria, which dynamics and turnover are crucial for cellular homeostasis and differentiation. Mitochondria form dynamic networks that are necessary for the maintenance of the organelle fidelity [58]. Various proteins participate in the regulation of mitochondrial dynamics, and a deregulation of mitochondrial dynamics is not only related to deregulation of mitochondrial function, but also closely correlated with apoptosis and, expectedly, linked to several diseases.

**Figure 2:**
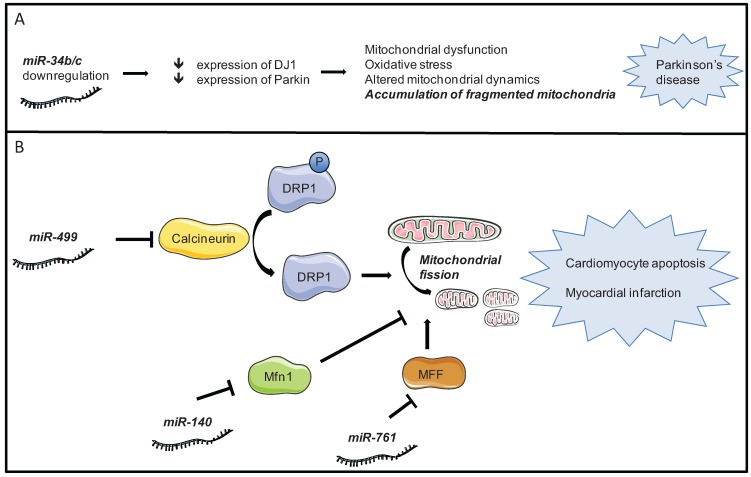
Schematic representation of mitochondrial fission modulation by miRNAs in disease. (**A**) In the brain, downregulation of miR-34b/c is associated with reduced expression of DJ1 and Parkin proteins, which in turn can lead to mitochondrial fragmentation and dysfunction, causing Parkinson’s disease; (**B**) In cardiomyocytes, the modulation of mitochondrial fusion/fission proteins lead to mitochondrial fragmentation and cardiomyocyte apoptosis/myocardial infarction. The expression of miR-499 or miR-761 and the downregulation of miR-140 could prevent mitochondrial fission and apoptosis. DRP1, dynamin-related protein-1; Mfn1, mitofusin 1; MFF, mitochondrial fission factor.

It has been found that some miRNAs can also affect mitochondrial dynamics. For example miR-761 is responsible for the downregulation of the mitochondrial fission factor (MFF) and it can suppress mitochondrial fission and apoptosis by targeting MFF (Figure 2), demonstrating that miR-761 and MFF constitute a regulatory axis in the machinery of the mitochondrial network and apoptosis [59]. Other studies have also revealed the modulation of mitochondrial fission through miRNA action; miR-30 family members have been reported to regulate apoptosis by targeting the mitochondrial fission machinery, inhibiting mitochondrial fission by suppressing the expression of p53 and its downstream target Drp-1 [60]. Figure 2 depicts the involvement of mitochondrial dynamics, specifically mitochondrial fission, in disease development and its modulation by miRNAs.

Mitochondrial fission and its modulation by miRNA has also been shown in some metabolic conditions; miR-484 downregulation may contribute to the pathogenesis of Insulin resistance by targeting mitochondrial fission protein 1 (Fis1), which is needed for mitochondrial fission [61], as mitochondrial fission is increased in diabetes and contributes to circulating insulin levels [62].

Certain tissues or cell types, such as cardiomyocytes, are particularly enriched in mitochondria, and this renders them more susceptible to be affected by mitochondrial dynamics alterations. Specifically, growing evidence has been linking abnormal mitochondrial fission with the initiation of apoptosis. Both mitochondrial fission and apoptosis are complex processes, controlled by a large number of proteins. It has been revealed that Mitofusin 1 (Mfn1), a mitochondrial fusion protein, is able to prevent mitochondrial fission [63]. Moreover, miRNA contribution to the regulation of the process has already been shown; Mfn1 is downregulated by apoptotic stimulation and miR-140 is upregulated, contributing to the suppression of Mfn1 expression during apoptosis. Thus, miR-140 can promote mitochondrial fission and apoptosis by targeting Mfn1 [63], ultimately causing cardiomyocyte apoptosis and infarction (Figure 2).

Apoptosis is essential for normal development and maintenance of tissue homeostasis. However, excessive apoptosis can occur in several tissues, causing cellular dysfunction and tissue-specific damage and pathology. In the myocardium, apoptosis contribute to the pathogenesis of cardiac diseases. A variety of stimuli can trigger apoptosis in cardiomyocytes, such as ROS [64], hypoxia [65], and anthracyclines [66]. Apoptosis is controlled by a complex interplay between pro- and anti-apoptotic factors. Under physiological conditions, this interplay remains in equilibrium so that apoptosis is tightly controlled. However, some clues on the regulation of mitochondrial dynamics and apoptosis by miRNAs have already been revealed. Some authors have shown that miR-30 family members are able to inhibit mitochondrial fission and apoptosis by suppressing the expression of p53 that can upregulate Drp1 [67]. Additionally, the mitochondrial network is controlled by a variety of proteins, for which some authors have already reported interactions of miRNAs; namely, the interaction of miRNA with Drp1 that can inhibit hydrogen peroxide-induced cell death [60]. In the heart, it has also been shown that miRNA is integrated into the mitochondrial network by controlling Mfn1. The axis of miR-140 and Mfn1 (Figure 2) can be a therapeutic target for the development of interventional treatment of mitochondrion-related cardiac diseases [63].

The removal of damaged mitochondria by autophagy, a process referred to as mitophagy, is critical for maintaining proper cellular functions. Mitophagy regulates the number of mitochondria to match the metabolic or developmental demands [68], and is also a part of the quality control pathway based on the removal of impaired mitochondria [69]. Several studies have suggested a role for miRNAs in autophagy, including miR-101 [70], miR-204 [71] and miR-30a [72]. miRNAs target transcripts of autophagy-related proteins, thereby suppressing their function in autophagy with pathological consequences [73]. For example, down-regulation of miR-34b/c is an early event in Parkinson’s disease [74], concomitantly with a reduced expression of DJ1 and Parkin proteins that correlate with increased mitochondrial fission and mitochondrial dysfunction (Figure 2). Besides, mitophagy has been characterized as a HIF-dependent mechanism [75], and hypoxic repression of the mitochondrial function by miR-210 may trigger mitophagy. The Table 2 presents a list of some miRNAs known to be involved in the regulation of the autophagy mechanism, by targeting some of the key players involved in the process.

**Table 2:** Example of some miRNAs involved in the regulation of autophagy.

miRNA	Target	Effect on autophagy	Reference
miR-101	STMN1, RAB5A and ATG4D	Inhibitor	[70]
miR-204	LC3	Inhibitor	[71]
miR-30a	Beclin 1	Inhibitor	[72,76]
miR-137	NIX, FUNDC1	Inhibitor (mitophagy)	[77]

Mitochondria are intimately involved in the aging process as well. Mitochondrial dysfunction is associated with the aging process and with the pathogenesis of a variety of disorders such as metabolic syndrome and neurodegenerative diseases [78]. In normal conditions, damaged or dysfunctional mitochondria are removed via autophagy and restored by fusion with healthy elements of the mitochondrial network. However, during aging autophagy declines and fission outpaces fusion [79], leading to accumulation of dysfunctional mitochondria. These produce large amounts of ROS and induce oxidative damage as well as enhanced mitochondrial permeability, leading either to apoptosis [80]. The decline of autophagic clearance during aging affects the equilibrium between mitochondrial fusion and fission, leading to a build-up of dysfunctional mitochondria, oxidative stress, chronic low-grade inflammation, and increased apoptosis rates, the main hallmarks of aging. Current research suggests that a large number of miRNAs are differentially expressed during cell aging [81], namely some of the referred mitomiRs (let7b, mir-146a, -133b, -106a, -19b, -20a, -34a, -181a and -221). Importantly, some algorithm analyses of aging-related mitomiRs targets have disclosed a number of resident mitochondrial proteins playing large roles in energy metabolism, mitochondrial transport and apoptosis [81]. Among these, Bcl-2 family members—which are critically involved in maintaining mitochondrial integrity—may play a role in controlling mitochondrial function and dysfunction during cellular aging, also considering that Bcl-2, the master member of the family, is an anti-oxidant and anti-apoptotic factor and regulates mitochondrial fission/fusion and autophagy. Thus, several aging-related mitomiRs may play a direct role in controlling mitochondrial function by regulating mitochondrial protein expression, hence their modulation could mediate the loss of mitochondrial integrity and function in aging cells, inducing or contributing to age-related diseases [81].

### 4.3. Cancer

**Figure 3:**
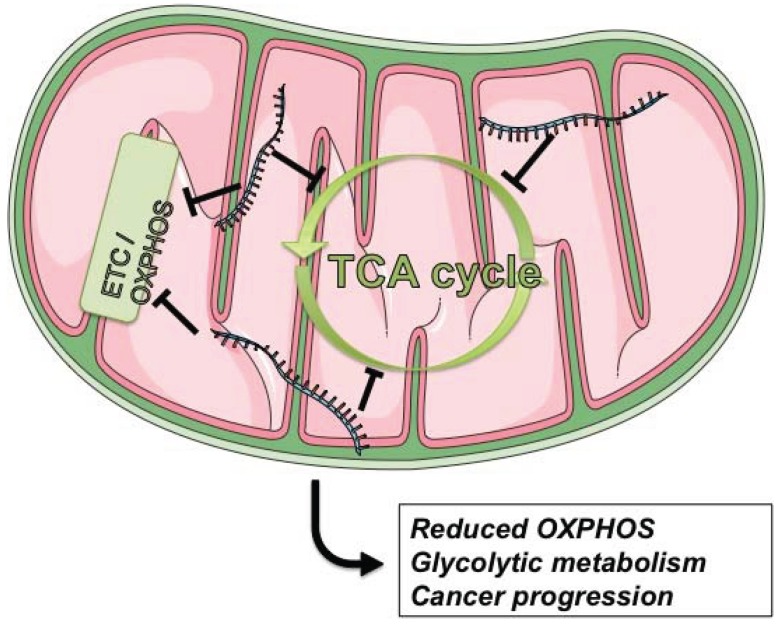
The action of oncogenic miRNAs in mitochondria. By targeting components involved in ETC/oxidative phosphorylation (OXPHOS) and in TCA cycle, miRNAs induce mitochondrial metabolic alterations and a shift from oxidative to glycolytic metabolism, ultimately contributing to Warburg effect and cancer progression.

Under aerobic conditions, the normal cells generate ATP primarily in mitochondrial OXPHOS, which utilizes products of glycolysis and Krebs cycle. Under anaerobic conditions, relative little pyruvate, the end product of glycolysis, is directed to the Krebs cycle and it is converted to lactate instead. Nevertheless, this metabolic conversion of glucose appears to be energetically disadvantageous. Interestingly, it was observed that many cancer cells prefer glycolysis instead of OXPHOS even in the presence of sufficient amount of oxygen. This anomalous energy metabolism is known as the Warburg effect [82,83]. Although the exact causes and functional consequences of this metabolic switch remain to be elucidated, there is a growing consensus that the Warburg effect is not an inconsequential byproduct of carcinogenesis, but is vital for cancer cells to maintain their proliferative potential, and is driven by various factors, including miRNAs [83,84]. In Figure 3 it is represented the overall action of some oncogenic miRNAs on mitochondrial ETC and TCA cycle.

In the last decade, miRNAs have emerged as critical regulators in cancer-related processes. Studies have shown that tumor-targeting therapies using miRNAs is becoming a novel diagnostic and therapeutic tool [85]. In particular, miR-200a has been demonstrated to suppress tumor growth in liver cancer [86], and also exhibited significantly lower expression in breast cancer [87], thus acting as a negative regulator or tumor suppressor for the cell growth, which is consistent with the role of miR-200a in hepatocellular carcinoma [88]. Several targets of miR-200a have been identified [89,90]. Based on bioinformatics analysis, TFAM was predicted as one of miR-200a targets. TFAM is the primary transcription factor in mitochondria [91], being also implicated as a primary architectural protein of the mitochondrial genome by packing the mtDNA and a key regulator involved in mtDNA transcription and replication [92]. Furthermore, it was reported that TFAM expression is involved in tumor progression, cancer cell growth, and chemoresistance [93]. Accordingly, TFAM has been reported as a functional target of miR-200a in breast cancer cells. MiR-200a overexpression significantly downregulated TFAM by directly targeting the 3'UTR of TFAM mRNA [87]. However, overexpression of TFAM without 3'UTR could overcome the inhibition by miR-200a and rescue the expression of TFAM, which could reverse the inhibition of TFAM-mediated mtDNA copy number by miR-200a [87]. Furthermore, miR-494 has been found to modulate mitochondrial biogenesis by downregulating the TFAM and the nuclear transcription factor Forkhead box J3 (FOXJ3) during myocyte differentiation and skeletal muscle adaptation to physical exercise [94].

Deficiency in apoptosis is considered to be a major cause of the therapeutic resistance of several cancers, namely non small cell lung cancer (NSCLC) [95]. Apoptosis is activated and inactivated by a variety of genes, and it is well accepted that the response to cellular stress factors like DNA damage involves activation of tumor suppressor p53. The primary function of p53 is to activate transcription of genes that contain p53-binding sites in their promoters. Transcriptional targets of p53 include the proapoptotic B cell lymphoma-2 (Bcl-2) family member Bcl-2 associated X protein (Bax), which migrates to mitochondria from cytosol in response to apoptotic signals, permeabilizes the outer membrane, resulting in release of mitochondrial proteins such as cytochrome *c*, AIF, *etc.*, into the cytosol or nucleus where they are actively involved in the process of caspase activation and protein/DNA degradation [96]. miRNAs, as important regulators of gene expression, have been implicated in the regulation of critical processes that are deregulated in cancer cells, such as proliferation [97], differentiation [98] and apoptosis [99]. Among the miRNAs, miR-34 family members play important tumor suppressive roles, as they are directly regulated by p53 and compose the p53 network [100].

A recent report suggests that p53-induced miR-34a plays an important role in NSCLC sensitization in response to capsaicin-induced DNA damage [95]. Bcl-2 was identified as the miR-34a target in these capsaicin-treated NSCLC cells. Down-regulation of Bcl-2 created an anti-survival environment in these NSCLC cells that favored Bax-mediated apoptosis via mitochondrial death cascade involving caspase-9 and -3, but not caspase-8, thereby ruling out the involvement of extrinsic death pathway. It is also accepted that p53-target miR-34a inhibits SIRT1 thereby deacetylating and stabilizing p53 in a positive feed- back loop [101]. It is thus tempting to speculate that miR-34 may be an important player in modulating mitochondrial death pathway in cancer cells. Additionally, in some tumors the level of miR-21 is increased, and it suppresses the expression of PTEN, which regulates the mitophagy-associated PINK1 [102].

In the latest years, microRNA-126 (miR-126), an endothelial-specific miRNA located within intron 7 of epidermal growth factor-like domain 7 (EGFL7), has been demonstrated to act as a tumor suppressor in various types of human cancers such as oral squamous cell carcinoma (OSCC) [103], bladder cancer [104], lung cancer [105] or colorectal cancer (CRC) [106]. The expression of miR-126 is significantly decreased in OSCC tissues, when compared with that in their matched adjacent tissues, whereas the protein expression of EGFL7 is upregulated in OSCC tissues. The overexpression of miR-126 significantly reduced the protein expression of EGFL7 in OSCC cells; furthermore, transfection with a miR-126 mimic markedly suppressed cell proliferation, cell cycle progression, cell invasion and colony formation, while inducing cell apoptosis, which contrasted with the effects of transfection with an miR-126 inhibitor, demonstrating that miR-126 acts as a tumor suppressor and that it may so serve as a promising candidate for the treatment of OSCC [103]. It has also been found that miR-126 overexpression inhibits cell proliferation, migration and invasion, and induces cell arrest in the G0/G1 phase of CRC cells. Furthermore, it was demonstrated that miR-126-mediated tumor suppression might be partly dependent on Akt and ERK1/2 signaling pathways, revealing a novel target for therapeutic strategies in cancer [106].

Recently, it has been demonstrated that miR-126 also affects mitochondrial function and mitochondrial metabolism in cancer cells, resulting in tumor suppression of malignant mesothelioma (MM) by inducing cancer metabolic reprogramming [107].

miR-126 is frequently lost in many types of cancer. However, this seems to be a cell type-dependent event, given that stimuli such as mtDNA depletion or hypoxia induce an increase in miR-126 in nonmalignant cells but downregulate miR-126 in MM cells [107]. In the same study, it was shown that miR-126 represses Akt activation as a down-stream effector of the adaptor protein IRS1 (insulin receptor substrate-1), a direct target of miR-126 [108]. Therefore, it is postulated that miR-126 may affect the mitochondrial citrate metabolism by inhibiting the Akt pathway to restore the TCA cycle for the synthesis of ATP via OXPHOS. Ectopic expression of miR-126 elevated citrate levels by inhibiting ATP-citrate lyase (ACL), consequently favoring glucose oxidation and production of cellular energy rather than converting it to other macromolecules for cellular biosynthesis. Moreover, miR-126 affects mitochondrial energy metabolism, reduces mitochondrial respiration, and promotes glycolysis in MM cells. This metabolic shift, associated with IRS1-modulated ACL deregulation, results in higher ATP and citrate production. This metabolic reprogramming allow a loss of malignancy, lack of anchorage-independent growth and suppression of tumor initiation and promotion, all results of an increased level of miR-126 [107].

The ever growing evidences of the involvement of miRNAs in cancer progression and invasion is supporting the emergent use of miRNAs for clinical assessment of cancer aggressiveness and tumor biomarkers, as well as turning miRNAs in promising molecules for therapeutic anti-cancer approaches.

## 5. Conclusions

It has been clearly reported that miRNAs play a critical role in regulating mitochondrial function, either under physiological and pathological conditions. Mitochondria represent an essential center of physiological processes of the cell, whose deregulation largely contributes to the development and progression of diseases. This regulation can be achieved either by targeting nuclear-encoded mRNAs or mitochondrial-encoded mRNAs. However, there is still the need to better and fully clarify the rapport between an abnormal distribution of miRNAs, explicitly in mitochondria, and mitochondrial dysfunction or pathological conditions related to mitochondrial dysfunction.

Further knowledge about the specific functional consequence of modulating miRNA levels may eventually lead to miRNAs-based therapy for the treatment of mitochondria-related diseases, highlighting these post-transcriptional gene regulators as auspicious therapeutic targets.
